# Learning gaps among statistical competencies for clinical and translational science learners

**DOI:** 10.1017/cts.2020.498

**Published:** 2020-06-19

**Authors:** Robert A. Oster, Katrina L. Devick, Sally W. Thurston, Joseph J. Larson, Leah J. Welty, Paul J. Nietert, Brad H. Pollock, Gina-Maria Pomann, Heidi Spratt, Christopher J. Lindsell, Felicity T. Enders

**Affiliations:** 1 Department of Medicine, Division of Preventive Medicine, University of Alabama at Birmingham, Birmingham, AL, USA; 2 Division of Biomedical Statistics & Informatics, Department of Health Sciences Research, Mayo Clinic, Scottsdale, AZ, USA; 3 Department of Biostatistics and Computational Biology, University of Rochester, Rochester, NY, USA; 4 Division of Biomedical Statistics & Informatics, Department of Health Sciences Research, Mayo Clinic, Rochester, MN, USA; 5 Department of Preventive Medicine – Biostatistics, Department of Psychiatry and Behavioral Sciences, Northwestern University, Chicago, IL, USA; 6 Department of Public Health Sciences, Medical University of South Carolina, Charleston, SC, USA; 7 Department of Public Health Sciences, University of California Davis School of Medicine, Davis, CA, USA; 8 Department of Biostatistics and Bioinformatics, Duke University, Durham, NC, USA; 9 Department of Preventive Medicine and Population Health, University of Texas Medical Branch, Galveston, TX, USA; 10 Department of Biostatistics, Vanderbilt University Medical Center, Nashville, TN, USA

**Keywords:** Statistical competency, biostatistics, clinical and translational science, research training, learning gaps

## Abstract

**Introduction:**

Statistical literacy is essential in clinical and translational science (CTS). Statistical competencies have been published to guide coursework design and selection for graduate students in CTS. Here, we describe common elements of graduate curricula for CTS and identify gaps in the statistical competencies.

**Methods:**

We surveyed statistics educators using e-mail solicitation sent through four professional organizations. Respondents rated the degree to which 24 educational statistical competencies were included in required and elective coursework in doctoral-level and master’s-level programs for CTS learners. We report competency results from institutions with Clinical and Translational Science Awards (CTSAs), reflecting institutions that have invested in CTS training.

**Results:**

There were 24 CTSA-funded respondents representing 13 doctoral-level programs and 23 master’s-level programs. For doctoral-level programs, competencies covered extensively in required coursework for all doctoral-level programs were basic principles of probability and hypothesis testing, understanding the implications of selecting appropriate statistical methods, and computing appropriate descriptive statistics. The only competency extensively covered in required coursework for all master’s-level programs was understanding the implications of selecting appropriate statistical methods. The least covered competencies included understanding the purpose of meta-analysis and the uses of early stopping rules in clinical trials. Competencies considered to be less fundamental and more specialized tended to be covered less frequently in graduate courses.

**Conclusion:**

While graduate courses in CTS tend to cover many statistical fundamentals, learning gaps exist, particularly for more specialized competencies. Educational material to fill these gaps is necessary for learners pursuing these activities.

## Introduction

Statistics are needed to summarize, analyze, and report on data obtained from a variety of research studies. A basic understanding of statistics is essential for investigators to understand the methods and results of these research studies, especially if they wish to translate the methods or results to other scientific research or clinical practice. Statistical analyses are often reported in the medical research literature [1–3], requiring investigators to have basic competencies in statistics to more fully comprehend the results [4,5]. In addition, there is a need to understand when statistical results have been incorrectly reported or when statistical methods have been incorrectly applied. Several authors have reported on statistical and research design problems in manuscripts that have been submitted to or have already been published in high-impact peer-reviewed journals [6–8]. Competency in statistics is also important for clinical and translational science (CTS) learners when consulting and collaborating with biostatisticians [9,10].

We define a CTS learner as a learner in a CTS program. CTS programs are funded by Clinical and Translational Science Awards (CTSAs) [11] and have been responsive to the National Center for Advancing Translational Sciences Requests for Applications (RFAs) and subsequent guidelines. By following the RFAs and guidelines, the intent and education goals of these CTS programs should be similar despite other differences that may exist among these programs.

Building on work done by the Education Key Function Committee of the CTSA National Consortium [12] and the Association of Schools of Public Health [13] and from established guidelines for the reporting of clinical and observational studies [14–17], we previously developed a comprehensive set of 24 statistical competencies for graduate coursework in the health sciences [4,5,12,18]. These competencies help define statistical topics that should be taught to health science learners of different types, including CTS learners. We have previously shown that not all CTS learners need detailed instruction on all competencies [4]. For example, learners who intend to become principal investigators need more training in fundamental, intermediate, and specialized competencies than those who intend to become informed readers of the medical literature [18]. However, of the 24 statistical competencies we identified, 19 were determined to be fundamental to general training for all CTS learners [4].

What is not known is the extent to which learners who are enrolled in a doctoral or master’s degree program in CTS, or in a similar degree program, are exposed to each of these statistical competencies. Our objective in this report is to therefore identify common statistical competency gaps so that they might be covered in future new educational content. Based on a survey of institutions with CTSAs, we present new information on the degree to which statistical competencies are covered in graduate education for CTS learners at both the master’s and doctoral levels. We identify competencies that are consistently taught, as well as those that are covered least often. In addition, we examine the relationship between how extensively competencies are covered and how fundamental they are perceived to be.

## Materials and Methods

This survey study was conducted from October 2019 through November 2019, with approval of the Mayo Clinic Institutional Review Board. To obtain a response from as many CTS institutions as possible in which statistics is taught to graduate CTS learners, we issued the survey through four professional organizations: (1) the Association of Clinical and Translational Science Biostatistics, Epidemiology, and Research Design Special Interest Group (BERD SIG) [19]; (2) the Association of Clinical and Translational Statisticians (ACTStat) [20]; (3) the American Statistical Association Section on Teaching of Statistics in the Health Sciences (TSHS) [21]; and (4) the American Statistical Association Section on Statistics and Data Science Education (EDUC) [22]. These are the groups that the authors believed would have the most experience with and be the most likely to teach CTS learners. At the time of the survey, the approximate membership of each of these groups was as follows: BERD SIG, 111; ACTStat, 128; TSHS, 650; and EDUC, 1300. Because the membership of these groups overlaps, it was not possible for us to determine how many members in each group or across all groups received the survey or which members would qualify to take the survey. While membership numbers are large, the groups also may include many members who are not part of CTSAs. Fifty of the 58 CTSAs currently have members in the BERD SIG.

Survey data were collected using Qualtrics software [23] (the survey instrument appears in the Supplementary Material). Email messages were sent to all members of the four organizations on days 0, 14, and 24. The survey was open for a total of five weeks. Each email message consisted of a cover letter and a link to the survey. The cover letter included instructions describing who should respond as well as information about ways in which the survey results might help course instructors and institutional graduate programs. Since our sampling methodology could potentially result in multiple submissions from a single institution, we stipulated that the primary respondent should be the person responsible for the first statistical course taught during that institution’s graduate program(s) in CTS that is(are) formally aligned with the institution’s CTSA. Respondents were asked to work with other instructors at their institutions of required or elective statistics courses available to the graduate CTS learners when completing the survey.

Respondents were first asked about institutional characteristics, including the types of CTS learners taught and the scope of the graduate programs in CTS. Next, respondents rated the extent to which each of the 24 statistical competencies was covered in the institution’s doctoral and master’s programs in CTS. The possible responses were given on a semi-quantitative 4-point Likert scale, with 1 representing “extensively covered in required courses,” 2 representing “briefly covered in required courses,” 3 representing “covered in elective courses only,” and 4 representing “not covered in any coursework.”

### Statistical Methods

Institutional characteristics, including information on course instructors and affiliated teaching staff, degrees offered, and the most advanced activity for which the programs are training their CTS learners, were summarized using descriptive statistics including frequencies and proportions. Among CTS programs, the proportion of doctoral and master’s curricula in which each of the competencies of interest was covered is similarly presented, along with graphical presentations including relative frequency bar plots.

To contextualize coverage of the competencies, we cross-referenced our data with a previous survey of 112 biostatisticians who graded each competency on whether or not they consider it to be fundamental to general training for CTS learners [4]. We define “percent fundamental” as the proportion of prior respondents who rated the competency as fundamental in the prior survey [4]. We evaluated the association between the percent fundamental from our prior work [4] and the percent each competency was covered in our current survey. The latter was defined as the proportion of current respondents indicating a competency was covered in a particular category of coursework. These associations were evaluated separately for doctoral and master’s programs using scatterplots and Spearman correlation coefficients (which are denoted using r_s_). R version 3.4.2 was used to perform all statistical analyses [24].

Because education of CTS learners is a central mission of the CTSA program, we were primarily interested in responses from CTSA-funded institutions [11]. However, because of our broad sampling of the four professional groups, we hoped to obtain responses from non-CTSA institutions who are also teaching CTS learners (e.g., institutions holding IDeA-CTR awards [25], which are similar to CTSAs but smaller in scope, or institutions that previously held a CTSA or IDeA award or have applied for such funding but have not received it). Due to the small number of responses from institutions not currently holding CTSA funding, we limited our statistical analysis of competency coverage of CTS programs only to CTSA-funded institutions. By narrowing the sampling frame for competency coverage of CTS programs to CTSA-funded institutions, competency coverage reported here reflects rates for institutions whose educational approaches are consistent with the goals of CTSA funding.

## Results

We received 32 responses. Of these, 24 (75%) were from CTSA-funded institutions, 1 (3%) was from an institution holding a funded IDeA-CTR grant, 3 (9%) had applied for a CTSA grant that was not currently awarded, and 4 (12%) were from institutions that had never applied for a CTSA or IDeA-CTR grant. During the survey response period, 58 institutions held funded CTSA grants [11], of whom 41% are represented in our results.

All of the respondents from CTSA-funded institutions reported that their institutions offered either a doctoral or master’s degree to CTS learners; 13 (54%) offer a doctoral degree (PhD, DrPH or ScD) to CTS learners, 16 (67%) offer a master’s degree (MS, MPH, MSCI, or MHS) with required prior doctoral degree (MD, PhD, etc.), and 18 (75%) offer a master’s degree to those without a prior doctoral degree. The number of students enrolled in the first required statistics course for CTS doctoral learners ranged from the category of 6–15 students to the category of 81 or more students, with a median category of 6–15 students. The mathematics/statistics background required for CTS doctoral learners prior to entering the CTS program varied across institutions. Respondents from five programs reported no prerequisites, respondents from five did not specify whether there were any prerequisites, and respondents from three reported prerequisites: one required applied statistics, introductory and multivariable calculus; one required multivariable calculus, mathematical statistics, and linear algebra; and the third required applied statistics, introductory and multivariable calculus, mathematical statistics, and probability.

Many types of CTS learners were enrolled in the statistics classes taught at the 24 institutions. In these classes, instructors at the majority of the institutions taught K scholars (*n* = 21), physicians (*n* = 21), CTS doctoral candidates (*n* = 15), other doctoral candidates (*n* = 23), Fellows (*n* = 20), and MS candidates (*n* = 20). Instructors at half of the institutions (*n* = 12) taught MPH candidates, while those at smaller numbers of institutions taught non-degree investigators (*n* = 10), research coordinators (*n* = 3), nurses (*n* = 9), chart abstractors (*n* = 1), and other learners (*n* = 4). Respondents reported that instructors who teach the first statistics course to CTS learners hold either a doctoral degree (*n* = 20) or master’s degree (*n* = 4). Their ranks were evenly split among Assistant (*n* = 6), Associate (*n* = 5), and Full Professor (*n* = 7), while 3 have the rank of Instructor, 2 have rank “other,” and 1 response was missing.

Respondents from 12 of the 13 doctoral programs reported that they were training CTS learners to lead research as a principal investigator (PI) and to help design future studies, while for one doctoral program, CTS learners were being trained to read and comprehend the medical research literature. Of the respondents from the master’s programs, 13/23 reported that they were training CTS learners to lead research as a PI and to help design future studies, 8/23 reported that the most advanced activity expected from their CTS learners was to read and comprehend the medical research literature, and 2/23 reported that they were training research staff.

**Fig. 1.**
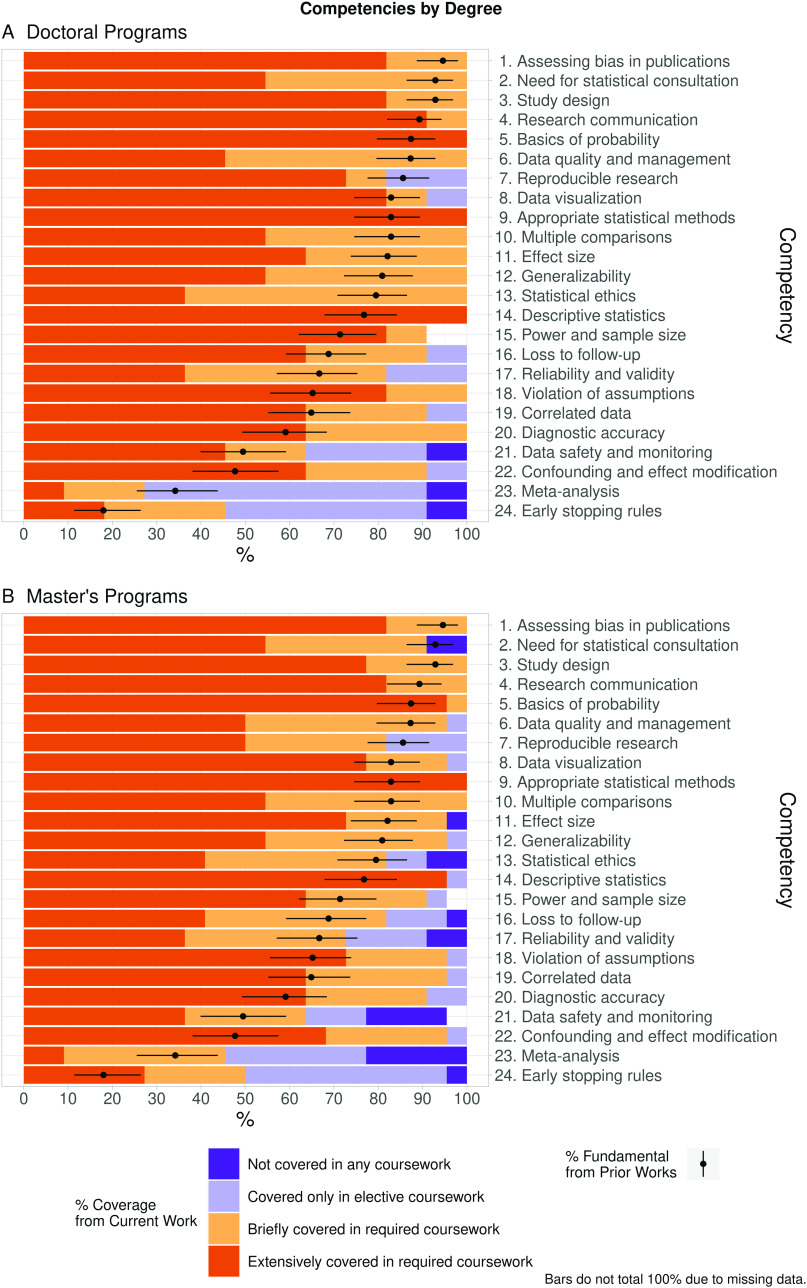
Coverage of each statistical competency in coursework for doctoral and master’s CTSA programs. For each of the 24 statistical competencies, the percentage of CTSA institutions that rated the competency as “extensively covered in required coursework,” “briefly covered in required coursework,” “covered in elective courses only,” or “not covered in any coursework” are displayed on the x-axis and plotted separately for doctoral (top: *n* = 11 CTSA programs) and master’s programs (bottom: *n* = 22 CTSA programs). The mean percent fundamental (with corresponding 95% CI) for each competency as reported in Enders et al. [4] is overlaid on each bar. The percentages for each bar may not sum to 100% due to missing data. Please see Table 1 for the corresponding frequencies and relative frequencies, in addition to a complete description of each competency.

Twenty one of the 24 (88%) CTSA-funded respondents answered competency-related questions for their doctoral and/or master’s programs. The competency ratings (“extensively covered in required coursework,” “briefly covered in required coursework,” “covered in elective coursework only,” or “not covered in any coursework”) for respondents in 11/13 (85%) doctoral programs and 22/23 (96%) master’s programs that answered competency-related questions are summarized in Fig. 1 and Table 1. For brevity and clarity, we refer to competencies by number and by shortened competency names throughout this text and in the figures; the numbers, full names, and shortened names are listed in Table 1.

**Table 1.** Competency frequencies and relative frequencies by degree program type for CTSA institutions, including full definitions and short competency names used in our manuscript

	Short competency name used in manuscript	Master’s programs (*N* = 23)	Doctoral programs (*N* = 13)
**Competency 1: Assess sources of bias and variation in published studies and threats to study validity (bias) including problems with sampling, recruitment, randomization, and comparability of study groups**	Assessing bias in publications		
Extensively covered in required coursework		18 (78.3%)	9 (69.2%)
Briefly covered in required coursework		4 (17.4%)	2 (15.4%)
Covered only in elective coursework		0 (0.0%)	0 (0.0%)
Not covered in any coursework		0 (0.0%)	0 (0.0%)
Missing		1 (4.3%)	2 (15.4%)
**Competency 2: Recognize limitation in statistical competency and realize when it would be best to involve a professional statistician**	Need for statistical consultation		
Extensively covered in required coursework		12 (52.2%)	6 (46.2%)
Briefly covered in required coursework		8 (34.8%)	5 (38.5%)
Covered only in elective coursework		0 (0.0%)	0 (0.0%)
Not covered in any coursework		2 (8.7%)	0 (0.0%)
Missing		1 (4.3%)	2 (15.4%)
**Competency 3: Identify the strengths and limitations of study designs for addressing a clinical or translational research question**	Study design		
Extensively covered in required coursework		17 (73.9%)	9 (69.2%)
Briefly covered in required coursework		5 (21.7%)	2 (15.4%)
Covered only in elective coursework		0 (0.0%)	0 (0.0%)
Not covered in any coursework		0 (0.0%)	0 (0.0%)
Missing		1 (4.3%)	2 (15.4%)
**Competency 4: Communicate research findings for scientific and lay audiences**	Research communication		
Extensively covered in required coursework		18 (78.3%)	10 (76.9%)
Briefly covered in required coursework		4 (17.4%)	1 (7.7%)
Covered only in elective coursework		0 (0.0%)	0 (0.0%)
Not covered in any coursework		0 (0.0%)	0 (0.0%)
Missing		1 (4.3%)	2 (15.4%)
**Competency 5: Understand the basic principles and practical importance of probability, random variation, commonly used statistical probability distributions, hypothesis testing, type I and type II errors, and confidence limits**	Basics of probability		
Extensively covered in required coursework		21 (91.3%)	11 (84.6%)
Briefly covered in required coursework		1 (4.3%)	0 (0.0%)
Covered only in elective coursework		0 (0.0%)	0 (0.0%)
Not covered in any coursework		0 (0.0%)	0 (0.0%)
Missing		1 (4.3%)	2 (15.4%)
**Competency 6: Understand the value of data quality and data management**	Data quality and management		
Extensively covered in required coursework		11 (47.8%)	5 (38.5%)
Briefly covered in required coursework		10 (43.5%)	6 (46.2%)
Covered only in elective coursework		1 (4.3%)	0 (0.0%)
Not covered in any coursework		0 (0.0%)	0 (0.0%)
Missing		1 (4.3%)	2 (15.4%)
**Competency 7: Understand the reasons for performing research that is reproducible from data collection through publication of results**	Reproducible research		
Extensively covered in required coursework		11 (47.8%)	8 (61.5%)
Briefly covered in required coursework		7 (30.4%)	1 (7.7%)
Covered only in elective coursework		4 (17.4%)	2 (15.4%)
Not covered in any coursework		0 (0.0%)	0 (0.0%)
Missing		1 (4.3%)	2 (15.4%)
**Competency 8: Understand appropriate methods for data presentation, especially effective statistical graphs and tables**	Data visualization		
Extensively covered in required coursework		17 (73.9%)	9 (69.2%)
Briefly covered in required coursework		4 (17.4%)	1 (7.7%)
Covered only in elective coursework		1 (4.3%)	1 (7.7%)
Not covered in any coursework		0 (0.0%)	0 (0.0%)
Missing		1 (4.3%)	2 (15.4%)
**Competency 9: Distinguish between variable types (e.g. continuous, binary, categorical) and understand the implications for selection of appropriate statistical methods**	Appropriate statistical methods		
Extensively covered in required coursework		22 (95.7%)	11 (84.6%)
Briefly covered in required coursework		0 (0.0%)	0 (0.0%)
Covered only in elective coursework		0 (0.0%)	0 (0.0%)
Not covered in any coursework		0 (0.0%)	0 (0.0%)
Missing		1 (4.3%)	2 (15.4%)
**Competency 10: Understand the potential misinterpretation of results in the presence of multiple comparisons**	Multiple comparisons		
Extensively covered in required coursework		12 (52.2%)	6 (46.2%)
Briefly covered in required coursework		10 (43.5%)	5 (38.5%)
Covered only in elective coursework		0 (0.0%)	0 (0.0%)
Not covered in any coursework		0 (0.0%)	0 (0.0%)
Missing		1 (4.3%)	2 (15.4%)
**Competency 11: Evaluate size of the effect with a measure of precision**	Effect size		
Extensively covered in required coursework		16 (69.6%)	7 (53.8%)
Briefly covered in required coursework		5 (21.7%)	4 (30.8%)
Covered only in elective coursework		0 (0.0%)	0 (0.0%)
Not covered in any coursework		1 (4.3%)	0 (0.0%)
Missing		1 (4.3%)	2 (15.4%)
**Competency 12: Understand issues relating to generalizability of a study, including sampling methods and the amount and type of missing data**	Generalizability		
Extensively covered in required coursework		12 (52.2%)	6 (46.2%)
Briefly covered in required coursework		9 (39.1%)	5 (38.5%)
Covered only in elective coursework		1 (4.3%)	0 (0.0%)
Not covered in any coursework		0 (0.0%)	0 (0.0%)
Missing		1 (4.3%)	2 (15.4%)
**Competency 13: Evaluate the impact of statistics on ethical research (e.g. an inadequate power calculation may mean it is unethical to ask subjects to consent to a study) and of ethics on statistical practice**	Statistical ethics		
Extensively covered in required coursework		9 (39.1%)	4 (30.8%)
Briefly covered in required coursework		9 (39.1%)	7 (53.8%)
Covered only in elective coursework		2 (8.7%)	0 (0.0%)
Not covered in any coursework		2 (8.7%)	0 (0.0%)
Missing		1 (4.3%)	2 (15.4%)
**Competency 14: Compute descriptive and simple inferential statistics appropriate for the data and research question**	Descriptive statistics		
Extensively covered in required coursework		21 (91.3%)	11 (84.6%)
Briefly covered in required coursework		0 (0.0%)	0 (0.0%)
Covered only in elective coursework		1 (4.3%)	0 (0.0%)
Not covered in any coursework		0 (0.0%)	0 (0.0%)
Missing		1 (4.3%)	2 (15.4%)
**Competency 15: Understand the components of sample size, power, and precision**	Power and sample size		
Extensively covered in required coursework		14 (60.9%)	9 (69.2%)
Briefly covered in required coursework		6 (26.1%)	1 (7.7%)
Covered only in elective coursework		1 (4.3%)	0 (0.0%)
Not covered in any coursework		0 (0.0%)	0 (0.0%)
Missing		2 (8.7%)	3 (23.1%)
**Competency 16: Understand the need to address loss to follow-up**	Loss to follow-up		
Extensively covered in required coursework		9 (39.1%)	7 (53.8%)
Briefly covered in required coursework		9 (39.1%)	3 (23.1%)
Covered only in elective coursework		3 (13.0%)	1 (7.7%)
Not covered in any coursework		1 (4.3%)	0 (0.0%)
Missing		1 (4.3%)	2 (15.4%)
**Competency 17: Understand the concepts and bias implications of reliability and validity of study measurements and evaluate the reliability and validity of measures**	Reliability and validity		
Extensively covered in required coursework		8 (34.8%)	4 (30.8%)
Briefly covered in required coursework		8 (34.8%)	5 (38.5%)
Covered only in elective coursework		4 (17.4%)	2 (15.4%)
Not covered in any coursework		2 (8.7%)	0 (0.0%)
Missing		1 (4.3%)	2 (15.4%)
**Competency 18: Evaluate potential violations of the assumptions behind common statistical methods**	Violation of assumptions		
Extensively covered in required coursework		16 (69.6%)	9 (69.2%)
Briefly covered in required coursework		5 (21.7%)	2 (15.4%)
Covered only in elective coursework		1 (4.3%)	0 (0.0%)
Not covered in any coursework		0 (0.0%)	0 (0.0%)
Missing		1 (4.3%)	2 (15.4%)
**Competency 19: Identify when clustered, matched, paired, or longitudinal statistical methods must be used**	Correlated data		
Extensively covered in required coursework		14 (60.9%)	7 (53.8%)
Briefly covered in required coursework		7 (30.4%)	3 (23.1%)
Covered only in elective coursework		1 (4.3%)	1 (7.7%)
Not covered in any coursework		0 (0.0%)	0 (0.0%)
Missing		1 (4.3%)	2 (15.4%)
**Competency 20: Understand the concepts of sensitivity, specificity, positive and negative predictive value, and receiver operating characteristic curves**	Diagnostic accuracy		
Extensively covered in required coursework		14 (60.9%)	7 (53.8%)
Briefly covered in required coursework		6 (26.1%)	4 (30.8%)
Covered only in elective coursework		2 (8.7%)	0 (0.0%)
Not covered in any coursework		0 (0.0%)	0 (0.0%)
Missing		1 (4.3%)	2 (15.4%)
**Competency 21: Understand the purpose of data and safety monitoring plans**	Data safety and monitoring		
Extensively covered in required coursework		8 (34.8%)	5 (38.5%)
Briefly covered in required coursework		6 (26.1%)	2 (15.4%)
Covered only in elective coursework		3 (13.0%)	3 (23.1%)
Not covered in any coursework		4 (17.4%)	1 (7.7%)
Missing		2 (8.7%)	2 (15.4%)
**Competency 22: Identify appropriate methods to address potential confounding and effect modification**	Confounding and effect modification		
Extensively covered in required coursework		15 (65.2%)	7 (53.8%)
Briefly covered in required coursework		6 (26.1%)	3 (23.1%)
Covered only in elective coursework		1 (4.3%)	1 (7.7%)
Not covered in any coursework		0 (0.0%)	0 (0.0%)
Missing		1 (4.3%)	2 (15.4%)
**Competency 23: Understand the purpose of meta-analysis and its place in the hierarchy of evidence**	Meta-analysis		
Extensively covered in required coursework		2 (8.7%)	1 (7.7%)
Briefly covered in required coursework		8 (34.8%)	2 (15.4%)
Covered only in elective coursework		7 (30.4%)	7 (53.8%)
Not covered in any coursework		5 (21.7%)	1 (7.7%)
Missing		1 (4.3%)	2 (15.4%)
**Competency 24: Understand the uses, importance, and limitations of early stopping rules in clinical trials**	Early stopping rules		
Extensively covered in required coursework		6 (26.1%)	2 (15.4%)
Briefly covered in required coursework		5 (21.7%)	3 (23.1%)
Covered only in elective coursework		10 (43.5%)	5 (38.5%)
Not covered in any coursework		1 (4.3%)	1 (7.7%)
Missing		1 (4.3%)	2 (15.4%)

Three competencies were covered extensively in required courses for all doctoral programs: competencies 5 (basics of probability), 9 (appropriate statistical methods), and 14 (descriptive statistics). An additional 12 competencies (1–4, 6, 10–13, 15, 18, and 20) were covered either extensively or briefly by instructors of required courses in all institutions with doctoral programs that provided responses. For master’s programs, competency 9 was also covered extensively in required courses by instructors of all master’s programs that answered these questions (96%), and an additional 5 competencies (1, 3–5, and 10) were covered extensively or briefly in all master’s programs.

For both doctoral and master’s programs, competencies 23 (meta-analysis) and 24 (early stopping rules) were the least well covered, with instructors at the majority of institutions covering this material only in elective courses or not covering it in any coursework.

The degree to which each competency is covered in required or elective coursework is shown in Fig. 1. Overlaid in these bars is the percent fundamental (the proportion of prior respondents who rated the competency as fundamental, as described by Enders et al. [4]) and 95% confidence interval (CI) for each competency. For the doctoral programs (Fig. 1A), the percent that competencies 7 (reproducible research) and 23 (meta-analysis) were covered in required coursework (either briefly or extensively) was less than the percent fundamental for the corresponding competency. For the master’s programs (Fig. 1B), the percent that competencies 2 (need for statistical consultation) and 6 (data quality and data management) were covered in required coursework was less than the percent fundamental. This suggests that these competencies were perceived to be more fundamental than the extent to which they were actually covered in required doctoral or master’s coursework for CTS learners.

**Fig. 2.**
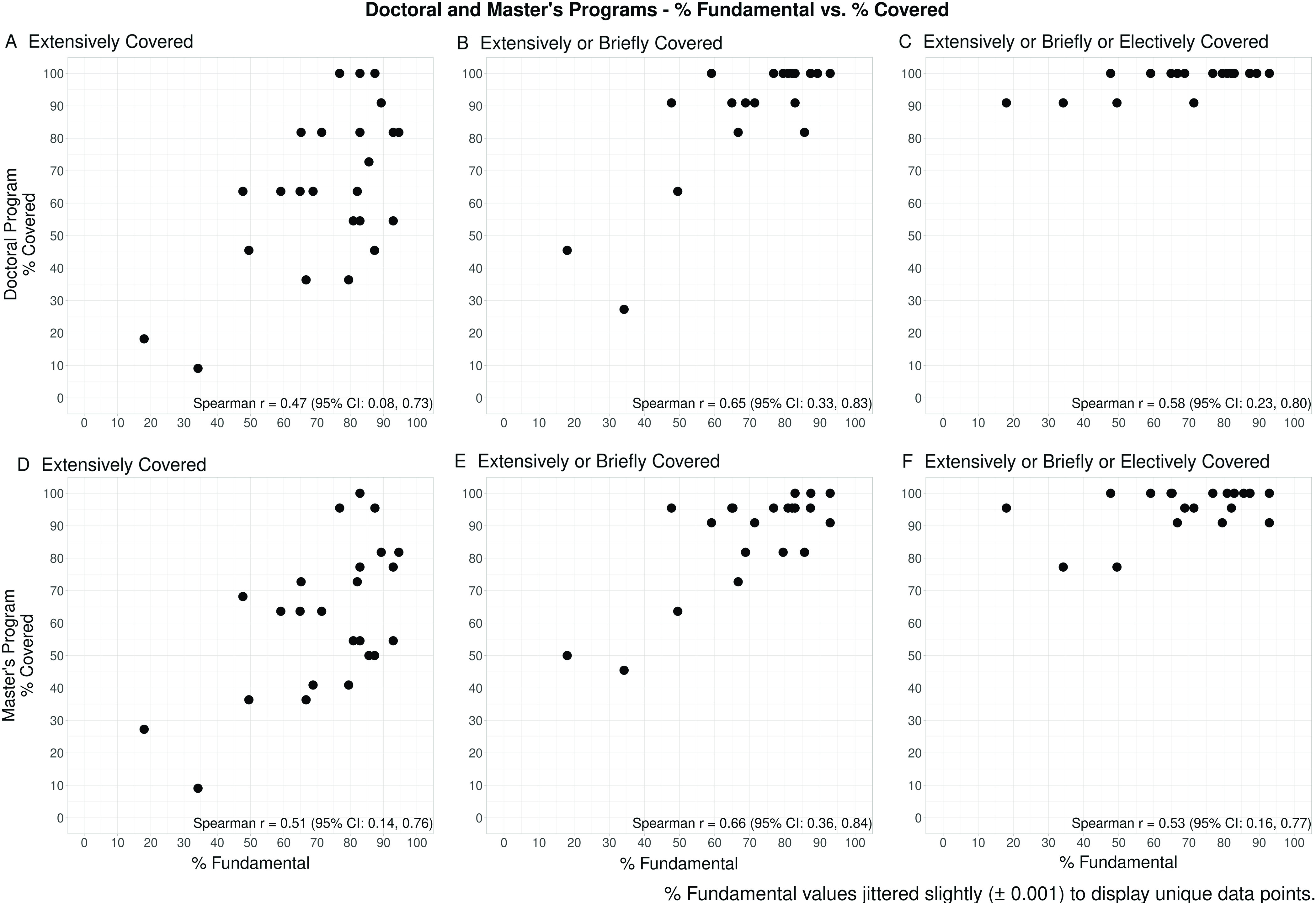
Relationship between the percent each of the 24 statistical competencies are covered in CTSA programs versus the percent fundamental. The percentage of CTSA programs that cover each competency (1) extensively in required coursework (left two panels), (2) extensively OR briefly in required coursework (middle two panels), or (3) extensively OR briefly in required coursework OR in elective coursework only (right two panels) is plotted versus the extent to which a competency was perceived as fundamental in prior work [4], separately for doctoral (top three panels: *n* = 11 CTSA programs) and master’s programs (bottom three panels: *n* = 22 CTSA programs). The 24 statistical competencies are represented by a dot in each graph. Spearman correlation coefficients and 95% confidence intervals are included on each plot.

To understand whether the competencies considered by biostatisticians as the most fundamental receive the most attention in graduate programs for CTS learners, we display the percent fundamental from Enders et al. [4] by the degree to which the corresponding competency is covered. The latter corresponds to collapsing over adjacent categories, specifically “extensively covered in required coursework,” “extensively covered in required coursework OR briefly covered in required coursework,” and “extensively covered in required coursework OR briefly covered in required coursework OR covered in elective coursework only,” and are shown separately for doctoral and master’s programs in Fig. 2. Considering the relationship between percent fundamental and “extensively covered in required coursework” (Fig. 2 A and D), we observed a positive relationship for both types of programs (*r*_*s*_ = 0.47 (95% CI: 0.08, 0.73) for doctoral and *r*_*s*_ = 0.51 (95% CI: 0.14, 0.76) for master’s programs), demonstrating that the higher the percent fundamental, the higher the percent a competency was covered in required coursework. Considering the relationship between percent fundamental and “extensively covered in required coursework OR briefly covered in required coursework” (Fig. 2B and E), we see that this positive relationship for both types of programs is strengthened (*r*_*s*_ = 0.65 (95% CI: 0.33, 0.83) for doctoral and *r*_*s*_ = 0.66 (95% CI: 0.36, 0.84) for master’s programs). However, this positive relationship for both types of programs is weaker when considering the relationship between the percent fundamental and “extensively covered in required coursework OR briefly covered in required coursework OR covered in elective coursework only” (Fig. 2C and F) (*r*_*s*_ = 0.58 (95% CI: 0.23, 0.80) for doctoral and *r*_*s*_ = 0.53 (95% CI: 0.16, 0.77) for master’s programs).

## Discussion

Through our survey of statistical educators, we have shown that graduate programs for CTS learners generally include the more fundamental statistical competencies. Our work also suggests that programs are generally comparable across the CTSA-funded institutions in terms of material covered.

We found that three of the competencies that were generally thought by statistical educators to be fundamental to the training of CTS learners in Enders et al. [4] were also universally included in required doctoral program coursework and nearly always included in required master’s program coursework. These included competencies 5 (basics of probability), 9 (appropriate statistical methods), and 14 (descriptive statistics). However, numerous competencies thought by biostatisticians to be fundamental [4] are extensively covered in required coursework in only 35% to 50% of graduate programs. These included competencies 2 (need for statistical consultation), 10 (multiple comparisons), 12 (generalizability), 13 (statistical ethics), and 17 (reliability and validity). For doctoral programs, these also included competency 11 (effect size). For master’s programs only, these also included competencies 7 (reproducible research) and 16 (loss to follow-up). Since these competencies comprise up to one-third of the 24 previously identified statistical competencies, they are important to recognize. We suggest that greater attention should be paid to these competencies (2, 7, 10, 11, 12, 13, 16, 17) and that graduate programs for CTS learners might need to fill the learning gaps by including these competencies in required coursework.

Competencies seen as less fundamental in Enders et al. [4] were less frequently included in the training of most CTS learners. These competencies include 21 (data safety and monitoring), 23 (meta-analysis), and 24 (early stopping rules). In our previous work [4], we specifically noted that these competencies are not fundamental for all learners although they are important for some, and we suggested that these competencies might be considered specialized statistical topics more appropriate for targeted training rather than for general graduate programs in CTS. We note that more than 20% of the statistical educators reported that instruction on data safety and monitoring and meta-analysis is not available in any master’s coursework and that nearly 10% of the statistical educators reported that instruction on early stopping rules (in clinical trials) is not available in any doctoral coursework.

Respondents from the majority (92%) of doctoral programs in CTS indicated that they are preparing CTS learners to be PIs of clinical and translational research studies, while those from 57% of master’s programs indicated that they are preparing CTS learners for this activity. Combined with results from our work that differentiate statistical concepts that are fundamental to all from those that are important for future PIs, this finding suggests an opportunity to customize training in master’s programs. We continue to emphasize that statistical competencies should not be taught using a “one size fits all” approach [18]. Ideally, all CTS learners, especially those who intend to become PIs, should have access to educational materials covering all statistical competencies. Competencies that are taught only in elective coursework may be less accessible since many elective courses are not offered annually. However, those who are training to become PIs may be motivated to take several elective courses. In some institutions, it is possible that a few of the competencies will not be available to CTS learners since these competencies are not taught in any coursework. In these situations, the learners may need to obtain training in these competencies at other institutions or perhaps in online workshops or one-on-one mentoring and education from statisticians or other faculty with requisite experience. These approaches may be especially helpful for the less fundamental competencies that align with specific specialized statistical topics within CTS, such as data and safety monitoring, meta-analysis, and early stopping rules (in clinical trials).

### Strengths and Limitations

In order to make the survey as representative as possible, we included the four national professional organizations for statisticians that the authors believed would have the most experience with and be the most likely to teach CTS learners, understanding that there was substantial overlap in membership among these groups, and that there would be some statistics educators of CTS learners who were not members of any of these groups.

Given the small number of respondents from non-CTSA-funded programs and our specific interest in CTSA-funded institutions, we limited our analysis of competency coverage to CTSA-funded institutions. Therefore, competency coverage reported in this paper is not representative of non-CTSA-funded institutions. Since statistics education for CTS learners is a fundamental goal of CTSA-funded institutions through their required workforce training and development components, competency coverage estimates presented here may reflect the best case (i.e., be among the highest) competency coverage rates in educational programs for health science researchers. We received responses from 24 out of 58 CTSA-funded institutions (41%). It is possible that responses from non-responding CTSA-funded institutions could differ systematically from the responses we received. Systematic differences that may exist between responding CTSA-funded institutions and non-responding CTSA-funded institutions include the size of the institution, the funding duration of the CTSA, and the number of survey respondents who were members of the BERD SIG. Unfortunately, we lack data to determine the degree to which the results from responding CTSA-funded institutions can be generalized to all CTSA-funded institutions.

Other limitations may include the following. Our survey results may have been solely based on one, or in some cases, multiple, statistics educator(s) responding on behalf of an institution. However, by requesting that respondents “please complete this survey in conjunction with others at your institution who teach the required statistical courses for doctoral and master’s degrees for your CTS program(s) or IDeA-CTR or similar programs,” we believe that the results obtained from a specific institution are not solely the view of one statistics educator. In addition, characteristics of the individual who teaches the first introductory statistics course may change from term to term and/or year to year. We do not view this as a major limitation since we believe that the content of the first introductory statistics course remains constant over time, even when there is a change in the instructor, in large part because the first course is often used as a prerequisite for other courses.

In our prior studies, we were not able to ascertain the specific number of respondents from each institution. An important strength of this study is that we have only a single collective response per institution, and in some cases, multiple relevant individuals at each institution agreeing upon the response data. It is possible that the team of individuals who responded may not be aware of all statistical education opportunities for CTS learners at their own institution, in which case competency coverage among CTSA-funded institutions could be larger than reported here.

## Conclusions

With the advent of the CTSAs, health science learners were brought into a new arena of research-related educational programs. Statistical competencies for these learners have evolved over time and will continue to evolve with new types of data and methodology to analyze these data. With our survey of these 24 statistical competencies, we observed that many of the most fundamental statistical concepts are extensively covered in courses taken by the majority of CTS learners. However, CTS learners who seek training in more specialized areas, such as clinical trials, do not always have access to coursework that focuses on all appropriate competencies. Opportunities exist for the development of more individualized coursework to cover competencies that are considered less fundamental, which may be especially relevant for CTS learners who intend to become PIs.

## Supplementary material

For supplementary material accompanying this paper visit https://doi.org/10.1017/cts.2020.498. click here to view supplementary material
