# Supplementary material for: Learning gaps among statistical competencies for clinical and translational science learners
Source: J Clin Transl Sci. 2020 Jun 19;5(1):e12. doi: 10.1017/cts.2020.498 (PMC8057376; doi:10.1017/cts.2020.498)
Supplement: Supplementary file 1 [file S2059866120004987sup001.pdf]

Q1

Learning Gaps for Statistical Competencies
for Clinical and Translational Science Learners

The following survey is intended to capture institutional level information about statistics coursework for institutions that offer a PhD or Master's degree and that currently hold CTSA or IDeA-CTR grants or institutions that have applied for or previously held one of these awards.

Please complete this survey in conjunction with others at your institution who teach the required statistical courses for doctoral and master's degrees for your Clinical and Translational Science (CTS) program(s) or IDeA-CTR or similar programs. We will be asking about the content of all your required statistical courses. Please consider the Doctoral and/or Master's degree programs you offer in Clinical and Translational Science or a closely related field. If you offer multiple programs or programs not in Clinical and Translational Science, please consider only those programs that are conceptually CLOSEST to Clinical and Translational Science, such as Public Health.

Thank you for agreeing to complete this survey on learning gaps for statistical competencies for CTS learners. This survey is in three parts. First, you will be asked for information about the people who teach statistics for CTS learners in your institution. Second, you will be asked to describe the selected training programs that you offer. Third, you will be asked about the statistical competencies that are included in your coursework.

DIRECTIONS:

Below is the link to the PDF version of the survey.

Please note that clicking on a link will open a new browser window/tab. To return to the survey, please navigate back to the survey window/ tab, or use your back button to return to the previous browser page.

Please <u>print</u> the PDF version of the survey and review it <u>prior</u> to completing the survey online. The survey BACK button will not be available on every page of the survey due to branching logic, so you will not be permitted to move back once you reach these portions of the survey.

- Learning Gaps Survey PDF

If at all possible, please send only one response per institution.

Please complete the survey in one sitting as responses will not save.

We would especially appreciate your responses by **Friday, November 22,** if at all possible.

If you have any questions while you are completing the survey, please contact one of the following individuals:

Robert Oster, PhD (roster@uabmc.edu)
Felicity Enders, PhD (Enders.Felicity@mayo.edu)
Sally Thurston, PhD (Sally_Thurston@URMC.Rochester.edu)

Q2

What is the name of your institution?

____

Q3

Does your institution currently hold a Clinical and Translational Science Award (CTSA) or Institutional Development Award for Clinical and Translational Research (IDeA-CTR) grant?

- ○ Yes, CTSA (1)
- ○ Yes, IDeA-CTR (2)
- ○ No, applied for CTSA, but not currently awarded (3)
- ○ No, applied for IDeA-CTR, but not currently awarded (4)
- ○ No, neither (5)

Q4

Please provide a link to your institution's degree programs relating to the prior question.

____

Q5

Please answer the following questions for the group of people who teach statistics for CTS learners at your institution.

Which of the following memberships do you as a group currently hold?
(Select all that apply.)

- ▢ American Statistical Association (ASA) https://www.amstat.org (1)
- ▢ APHA Applied Public Health Statistics https://apha.org/apha-communities/member-sections/applied-public-health-statistics (2)
- ▢ ASA Section on the Teaching of Statistics in the Health Sciences (TSHS) https://community.amstat.org/tshs/home (3)
- ▢ Association of Clinical and Translational Statisticians (ACTStat) http://actstat.org/ (4)
- ▢ The Biostatistics, Epidemiology, and Research Design (BERD) Special Interest Group in the Association of Clinical and Translational Science http://www.actscience.org/page/special-interest-groups-(sigs) (5)

Q6

Areas of the highest degrees of the people who teach statistics in your CTSA/ IDeA-CTR:
(Please provide the number of people in your group who's highest degree is in the following areas.)

________ Biostatistics (1)
________ Statistics (2)
________ Mathematics (3)
________ Epidemiology (4)
________ Other, please specify: (5) ____

Q7

Degree held by the person who teaches the <u>first</u> introductory statistics course to CTS learners:

- ○ Doctoral degree (1)
- ○ Master's degree (2)
- ○ Bachelor's degree (3)

Q8

Current academic appointments:

(Please provide the academic appointment of the person who teaches the first introductory statistics course to CTS learners.)

- ○ Teaching assistant (1)
- ○ Postdoctoral fellow (2)
- ○ Instructor (3)
- ○ Assistant Professor (4)
- ○ Associate Professor (5)
- ○ Professor (6)
- ○ Other, please specify: (7) ____

Q9

Please provide the gender of the person above.

- ○ Female (1)
- ○ Male (2)
- ○ Other (3)

Q10

In the last five years, which types of clinical and translational science (CTS) learners have you or other faculty in your CTSA/IDeA-CTR or potential CTSA/IDeA-CTR (if applicable) taught in your CTS courses? (Please select all that apply for your group.)

- ▢ K scholars (1)
- ▢ Physicians (2)
- ▢ CTS doctoral candidates (PhD, ScD, DrPH) (3)
- ▢ Other doctoral candidates (PhD, ScD, DrPH, PharmD, DVM, DDS, etc.) (4)
- ▢ Fellows (5)
- ▢ MS candidates (6)
- ▢ MPH candidates (7)
- ▢ Non-degree investigators (8)
- ▢ Research coordinators (9)
- ▢ Nurses (10)
- ▢ Chart abstractors (11)
- ▢ Other type of learners, please specify: (12) ____

Q11

What are the (abbreviated) degrees offered to CTS learners at your institution?
(Please select all that apply.)

- ▢ PhD, DrPH, or ScD (1)
- ▢ MS, MPH, MSCI, or MHS with **required prior doctoral degree (MD, PhD, etc.)** (2)
- ▢ MS, MPH, MSCI, or MHS with **NO required prior doctoral degree** (3)
- ▢ No doctoral or master’s degree offered (4)

Display This Question: If Q11 = 1

Q12

In your opinion, what is the most advanced activity for which you are training CTS learners in your Doctoral degree program aligned with your CTSA/IDeA-CTR or potential CTSA/IDeA-CTR (if applicable)? (Please provide a single answer for your institution.)

- ◯ To lead research as a PI and to help design future studies (1)
- ◯ To read and comprehend the medical research literature (2)
- ◯ To work as a research staff (3)

Display This Question: If Q11 = 2 Or Q11 = 3

Q13

In your opinion, what is the most advanced activity for which you are training CTS learners in your Master's degree program aligned with your CTSA/IDeA-CTR or potential CTSA/IDeA-CTR (if applicable)? (Please provide a single answer for your institution.)

- ◯ To lead research as a PI and to help design future studies (1)
- ◯ To read and comprehend the medical research literature (2)
- ◯ To work as a research staff (3)

Display This Question: If Q11 = 1 And If Q11 = 2 Or Q11 = 3

Q14

For both Doctoral and Master's degrees, are the required statistical courses identical for both programs?

- ◯ Required statistical courses are **identical** for our programs (1)
- ◯ Required statistical courses are **different** for our programs (2)

Q15

STOP!

Before you click "**Next >>**", you will not be able to go back and review or revise your previous answers. Please be sure you have downloaded a PDF copy of the survey and that your answers are correct/complete.

Please print the PDF version of the survey and review it prior to completing the rest of the online version.

- Learning Gaps Survey PDF

~ Thank you

**If Q11 =** 1............. complete Doctoral block	**If Q11 =** 1 *AND* 2 or 3 *AND* **Q14** = 1 .... complete Identical block
**If Q11 =** 2 or 3........complete Master's block	**If Q11 =** 1 *AND* 2 *AND* 3 *AND* **Q14** = 1 .... complete Identical block
**If Q11 =** 4............. skip to Q38	**If Q11 =** 1 *AND* 2 or 3 *AND* **Q14** = 2 .... complete Doctoral **AND** Master's block
	**If Q11 =** 1 *AND* 2 *AND* 3 *AND* **Q14** = 2 .... complete Doctoral **AND** Master's block

DOCTORAL Block Q16 - Q21

Complete if:

Q11 = 1

OR If Q11 = 1 AND 2 or 3 AND Q14 = 2

OR If Q11 = 1 AND 2 AND 3 AND Q14 = 2

Q16

Please complete the next questions for your PhD degree programs.

If you offer multiple programs or programs not in Clinical and Translational Science, please consider only those programs that are conceptually closest to Clinical and Translational Science, such as Public Health.

Q17

In what fields is this PhD degree offered?

(Select all that apply.)

- ☐ Clinical and Translational Science (1)
- ☐ Public Health (2)
- ☐ Other, please specify: (3) ____

Q18

How many total students (CTS plus other students) enroll annually in the first statistical course required for CTS PhD learners in your program?

- ○ 1 to 5 students (1)
- ○ 6 to 15 students (2)
- ○ 16 to 40 students (3)
- ○ 41 to 80 students (4)
- ○ 81 or more students (5)
- ○ N/A (not required statistical course) (6)

Q19

What is the required mathematics/statistics background of these CTS PhD learners prior to entering the CTS degree/training program? (Select all that apply.)

- ▢ Undergraduate applied statistics course (1)
- ▢ Undergraduate mathematical statistics course (2)
- ▢ Undergraduate probability course (3)
- ▢ Undergraduate introductory calculus course (at least 1 semester/quarter) (4)
- ▢ Undergraduate multivariable calculus course (5)
- ▢ Undergraduate linear algebra course (6)
- ▢ Other undergraduate mathematics course (please specify): (7) ________________________________
- ▢ None to my knowledge (8)

Q20

For each competency below, please select for your PhD degree program whether the competency is:

- Extensively covered by required coursework, such as the focus of a full lecture or included in multiple lectures and likely addressed in assessment.
- Briefly covered by required coursework, such as included in part of a lecture, with little or no assessment.
- Covered only in elective coursework.
- Not covered by any coursework (either required or elective).

Q21

What does the REQUIRED and ELECTIVE coursework in this PhD program include? (Please select the appropriate response in each row.)

	Extensively covered in required courses (1)	Briefly covered in required courses (2)	Covered in elective courses only (3)	Not covered in ANY coursework (4)
Assess sources of bias and variation in published studies and threats to study validity (bias) including problems with sampling, recruitment, randomization, and comparability of study groups (1)	○	○	○	○
Recognize limitation in statistical competency and realize when it would be best to involve a professional statistician (2)	○	○	○	○
Identify the strengths and limitations of study designs for addressing a clinical or translational research question (3)	○	○	○	○
Communicate research findings for scientific and lay audiences (4)	○	○	○	○
Understand the basic principles and practical importance of probability, random variation, commonly used statistical probability distributions, hypothesis testing, type I and type II errors, and confidence limits (5)	○	○	○	○
Understand the value of data quality and data management (6)	○	○	○	○
Understand the reasons for performing research that is reproducible from data collection through publication of results (7)	○	○	○	○
Understand appropriate methods for data presentation, especially effective statistical graphs and tables (8)	○	○	○	○
Distinguish between variable types (e.g. continuous, binary, categorical) and understand the implications for selection of appropriate statistical methods (9)	○	○	○	○
Understand the potential misinterpretation of results in the presence of multiple comparisons (10)	○	○	○	○
Evaluate size of the effect with a measure of precision (11)	○	○	○	○
Understand issues relating to generalizability of a study, including sampling methods and the amount and type of missing data (12)	○	○	○	○
Evaluate the impact of statistics on ethical research (e.g. an inadequate power calculation may mean it is unethical to ask subjects to consent to a study) and of ethics on statistical practice (13)	○	○	○	○

Compute descriptive and simple inferential statistics appropriate for the data and research question (14)	○	○	○	○
Understand the components of sample size, power, and precision (15)	○	○	○	○
Understand the need to address loss to follow-up (16)	○	○	○	○
Understand the concepts and bias implications of reliability and validity of study measurements and evaluate the reliability and validity of measures (17)	○	○	○	○
Evaluate potential violations of the assumptions behind common statistical methods (18)	○	○	○	○
Identify when clustered, matched, paired, or longitudinal statistical methods must be used (19)	○	○	○	○
Understand the concepts of sensitivity, specificity, positive and negative predictive value, and receiver operating characteristic curves (20)	○	○	○	○
Understand the purpose of data and safety monitoring plans (21)	○	○	○	○
Identify appropriate methods to address potential confounding and effect modification (22)	○	○	○	○
Understand the purpose of meta-analysis and its place in the hierarchy of evidence (23)	○	○	○	○
Understand the uses, importance, and limitations of early stopping rules in clinical trials (24)	○	○	○	○

Q22
STOP!
After you click "**Next >>**", you will not be able to go back and review or revise your previous answers.

MASTER'S Block Q22 – Q27

Complete if:

Q11 = 2 or 3

OR If Q11 = 1 AND 2 or 3 AND Q14 = 2

OR If Q11 = 1 AND 2 AND 3 AND Q14 = 2

Q23

Please complete the next questions for your Master's degree programs.

If you offer multiple programs or programs not in Clinical and Translational Science, please consider only those programs that are conceptually closest to Clinical and Translational Science, such as Public Health.

Q24

In what fields is the Master's degree offered?

(Select all that apply.)

▢ Clinical and Translational Science (1)

▢ Public Health (2)

▢ Other, please specify: (3) ____

Q25

How many total students (CTS plus other students) enroll annually in the first statistical course required for CTS Master's learners in your program?

○ 1 to 5 students (1)

○ 6 to 15 students (2)

○ 16 to 40 students (3)

○ 41 to 80 students (4)

○ 81 or more students (5)

○ N/A (no required statistical course) (6)

Q26

What is the required mathematics/statistics background of these CTS Master's learners prior to entering the CTS degree/training program?

(Select all that apply.)

- ☐ Undergraduate applied statistics course (1)
- ☐ Undergraduate mathematical statistics course (2)
- ☐ Undergraduate probability course (3)
- ☐ Undergraduate introductory calculus course (at least 1 semester/quarter) (4)
- ☐ Undergraduate multivariable calculus course (5)
- ☐ Undergraduate linear algebra course (6)
- ☐ Other undergraduate mathematics course (please specify): (7) ________________________________
- ☐ None to my knowledge (8)

Q27

For each competency below, please select for your Master's degree program whether the competency is:

- Extensively covered by required coursework, such as the focus of a full lecture or included in multiple lectures and likely addressed in assessment.
- Briefly covered by required coursework, such as included in part of a lecture, with little or no assessment.
- Covered only in elective coursework.
- Not covered by any coursework (either required or elective).

Q28

What does the REQUIRED and ELECTIVE coursework in this Master's program include? (Please select the appropriate response in each row.)

	Extensively covered in required courses (1)	Briefly covered in required courses (2)	Covered in elective courses only (3)	Not covered in ANY coursework (4)
Assess sources of bias and variation in published studies and threats to study validity (bias) including problems with sampling, recruitment, randomization, and comparability of study groups (1)	○	○	○	○
Recognize limitation in statistical competency and realize when it would be best to involve a professional statistician (2)	○	○	○	○
Identify the strengths and limitations of study designs for addressing a clinical or translational research question (3)	○	○	○	○
Communicate research findings for scientific and lay audiences (4)	○	○	○	○
Understand the basic principles and practical importance of probability, random variation, commonly used statistical probability distributions, hypothesis testing, type I and type II errors, and confidence limits (5)	○	○	○	○
Understand the value of data quality and data management (6)	○	○	○	○
Understand the reasons for performing research that is reproducible from data collection through publication of results (7)	○	○	○	○
Understand appropriate methods for data presentation, especially effective statistical graphs and tables (8)	○	○	○	○
Distinguish between variable types (e.g. continuous, binary, categorical) and understand the implications for selection of appropriate statistical methods (9)	○	○	○	○
Understand the potential misinterpretation of results in the presence of multiple comparisons (10)	○	○	○	○
Evaluate size of the effect with a measure of precision (11)	○	○	○	○
Understand issues relating to generalizability of a study, including sampling methods and the amount and type of missing data (12)	○	○	○	○
Evaluate the impact of statistics on ethical research (e.g. an inadequate power calculation may mean it is unethical to ask subjects to consent to a study) and of ethics on statistical practice (13)	○	○	○	○

Compute descriptive and simple inferential statistics appropriate for the data and research question (14)	○	○	○	○
Understand the components of sample size, power, and precision (15)	○	○	○	○
Understand the need to address loss to follow-up (16)	○	○	○	○
Understand the concepts and bias implications of reliability and validity of study measurements and evaluate the reliability and validity of measures (17)	○	○	○	○
Evaluate potential violations of the assumptions behind common statistical methods (18)	○	○	○	○
Identify when clustered, matched, paired, or longitudinal statistical methods must be used (19)	○	○	○	○
Understand the concepts of sensitivity, specificity, positive and negative predictive value, and receiver operating characteristic curves (20)	○	○	○	○
Understand the purpose of data and safety monitoring plans (21)	○	○	○	○
Identify appropriate methods to address potential confounding and effect modification (22)	○	○	○	○
Understand the purpose of meta-analysis and its place in the hierarchy of evidence (23)	○	○	○	○
Understand the uses, importance, and limitations of early stopping rules in clinical trials (24)	○	○	○	○

Q29
STOP!
After you click "**Next >>**", you will not be able to go back and review or revise your previous answers.

IDENTICAL Block 28 – Q34
Complete if:
Q11 = 1 AND 2 or 3 AND Q14 = 1
OR If Q11 = 1 AND 2 AND 3 AND Q14 = 2

Q30
Please complete the next questions for your PhD/Master's degree programs.

If you offer multiple programs or programs not in Clinical and Translational Science, please consider only those programs that are conceptually closest to Clinical and Translational Science, such as Public Health.

Q31
In what fields is the PhD degree offered?
(Select all that apply.)

- ▢ Clinical and Translational Science (1)
- ▢ Public Health (2)
- ▢ Other, please specify: (3) ____

Q32
In what fields is the Master's degree offered?
(Select all that apply.)

- ▢ Clinical and Translational Science (1)
- ▢ Public Health (2)
- ▢ Other, please specify: (3) ____

Q33

How many total students (CTS plus other students) enroll annually in the first statistical course required for CTS PhD/Master's learners in your program?

- ◯ 1 to 5 students (1)
- ◯ 6 to 15 students (2)
- ◯ 16 to 40 students (3)
- ◯ 41 to 80 students (4)
- ◯ 81 or more students (5)
- ◯ N/A (no required statistical course) (6)

Q34

What is the required mathematics/statistics background of these CTS PhD/Master's learners prior to entering the CTS degree/training program? (Select all that apply.)

- ▢ Undergraduate applied statistics course (1)
- ▢ Undergraduate mathematical statistics course (2)
- ▢ Undergraduate probability course (3)
- ▢ Undergraduate introductory calculus course (at least 1 semester/quarter) (4)
- ▢ Undergraduate multivariable calculus course (5)
- ▢ Undergraduate linear algebra course (6)
- ▢ Other undergraduate mathematics course (please specify): (7) ________________________________
- ▢ None to my knowledge (8)

Q35

For each competency below, please select for your PhD/Master's degree program whether the competency is:

- Extensively covered by required coursework, such as the focus of a full lecture or included in multiple lectures and likely addressed in assessment.
- Briefly covered by required coursework, such as included in part of a lecture, with little or no assessment.
- Covered only in elective coursework.
- Not covered by any coursework (either required or elective).

Q36

What does the REQUIRED and ELECTIVE coursework in this PhD/Master's program include?

(Please select the appropriate response in each row.)

	Extensively covered in required courses (1)	Briefly covered in required courses (2)	Covered in elective courses only (3)	Not covered in ANY coursework (4)
Assess sources of bias and variation in published studies and threats to study validity (bias) including problems with sampling, recruitment, randomization, and comparability of study groups (1)	○	○	○	○
Recognize limitation in statistical competency and realize when it would be best to involve a professional statistician (2)	○	○	○	○
Identify the strengths and limitations of study designs for addressing a clinical or translational research question (3)	○	○	○	○
Communicate research findings for scientific and lay audiences (4)	○	○	○	○
Understand the basic principles and practical importance of probability, random variation, commonly used statistical probability distributions, hypothesis testing, type I and type II errors, and confidence limits (5)	○	○	○	○
Understand the value of data quality and data management (6)	○	○	○	○
Understand the reasons for performing research that is reproducible from data collection through publication of results (7)	○	○	○	○
Understand appropriate methods for data presentation, especially effective statistical graphs and tables (8)	○	○	○	○
Distinguish between variable types (e.g. continuous, binary, categorical) and understand the implications for selection of appropriate statistical methods (9)	○	○	○	○
Understand the potential misinterpretation of results in the presence of multiple comparisons (10)	○	○	○	○
Evaluate size of the effect with a measure of precision (11)	○	○	○	○
Understand issues relating to generalizability of a study, including sampling methods and the amount and type of missing data (12)	○	○	○	○
Evaluate the impact of statistics on ethical research (e.g. an inadequate power calculation may mean it is unethical to ask subjects to consent to a study) and of ethics on statistical practice (13)	○	○	○	○

Compute descriptive and simple inferential statistics appropriate for the data and research question (14)	○	○	○	○
Understand the components of sample size, power, and precision (15)	○	○	○	○
Understand the need to address loss to follow-up (16)	○	○	○	○
Understand the concepts and bias implications of reliability and validity of study measurements and evaluate the reliability and validity of measures (17)	○	○	○	○
Evaluate potential violations of the assumptions behind common statistical methods (18)	○	○	○	○
Identify when clustered, matched, paired, or longitudinal statistical methods must be used (19)	○	○	○	○
Understand the concepts of sensitivity, specificity, positive and negative predictive value, and receiver operating characteristic curves (20)	○	○	○	○
Understand the purpose of data and safety monitoring plans (21)	○	○	○	○
Identify appropriate methods to address potential confounding and effect modification (22)	○	○	○	○
Understand the purpose of meta-analysis and its place in the hierarchy of evidence (23)	○	○	○	○
Understand the uses, importance, and limitations of early stopping rules in clinical trials (24)	○	○	○	○

Q37
STOP!
After you click "**Next >>**", you will not be able to go back and review or revise your previous answers.

Q38

Please describe any issues or concerns you had in filling out this survey.

____

____

Q39

In the unlikely event that we have questions, who would be the best person for us to contact? Please enter their email address below.

____

____

Q40

THANK YOU FOR COMPLETING THE SURVEY!

Please click SUBMIT to record your answers.
